# Preclinical evaluation of cysteine protease-inhibitor aloxistatin (E64d) for heart failure therapy

**DOI:** 10.1007/s00109-026-02695-5

**Published:** 2026-06-25

**Authors:** Maria Jordan, Angelika Stucki-Koch, Maximilian Schinke, Lena Willmer, Ingrid Senne, Naisam Abbas, Marco Bentele, Dana Oldenkott, Nico Lachmann, Linda Feldbrügge, Resa Puffert, Josef Fangmann, Robert Ramm, Thomas Thum, Katherina Sewald, Kevin Schmidt, Jan Fiedler

**Affiliations:** 1 Fraunhofer Institute for Toxicology and Experimental Medicine ITEM, Hannover, Germany; 2 Fraunhofer Cluster of Excellence for Immune Mediated Diseases (CIMD), Hannover, Germany; 3 Institute of Molecular and Translational Therapeutic Strategies (IMTTS), Hannover Medical School, Hannover, Germany; 4 Department for Pediatric Pneumology, Allergology and Neonatology, Hannover Medical School, Hannover, Germany; 5 REBIRTH, Research Center for Translational and Regenerative Medicine, Hannover Medical School, Hannover, Germany; 6 Cluster of Excellence RESIST (EXC 2155), Hannover Medical School, Hannover, Germany; 7 Biomedical Research in Endstage and Obstructive Lung Disease Hannover (BREATH), Member of the German Center for Lung Research (DZL), Hannover Medical School, Hannover, Germany; 8 Department of General, Visceral and Transplant Surgery, Hannover Medical School, Hannover, Germany; 9 KRH Siloah Hospital, Hannover, Germany; 10 Department for Cardiac, Thoracic, Transplantation and Vascular Surgery, Hannover Medical School, Hannover, Germany

**Keywords:** Heart failure, Aloxistatin, Cardiac fibrosis, Extracellular matrix, Living myocardial slices

## Abstract

**Abstract:**

Heart failure (HF) affects over 64 million people worldwide causally linked to fibrotic scarring. None of the available cardiac drugs target fibrosis directly, underlining unmet clinical need for novel therapies. This study aimed to explore the therapeutic potential of the cysteine protease inhibitor aloxistatin as a repurposed drug candidate to combat fibrotic progression in predictive HF models. Aloxistatin reduced migratory and proliferative capacities of human cardiac fibroblasts (HCFs) derived from various HF backgrounds. Mechanistically, aloxistatin attenuated TGFβ1-induced pro-fibrotic signaling in cardiomyopathy-derived HCFs by inhibiting extracellular matrix organization-related gene expression and secretion of MMP2 und FN1, partially mediated through CAPN2 inhibition. Transcriptomic analysis of rat *ex vivo* myocardial slices revealed a pronounced suppression of inflammatory pathways. Anti-inflammatory effects of aloxistatin were further confirmed by reduced NFκB activity in reporter cells and inhibited HLA-DR expression in human iPSC-derived macrophages. Application of diverse preclinical cardiac HF models arguably underlined aloxistatin as a potential drug repurposing strategy by simultaneously counteracting myocardial inflammatory signaling and pro-fibrotic mechanisms. This preclinical study suggests aloxistatin therapy for translational use to attenuate cardiac remodeling and progression of heart failure.

**Graphical abstract:**

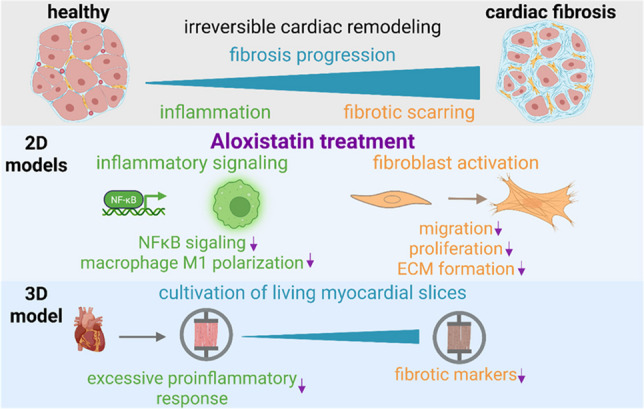

**Supplementary Information:**

The online version contains supplementary material available at 10.1007/s00109-026-02695-5.

## Key messages

- Calpain and cathepsin families are deregulated in heart failure progression.
- Calpain and cathepsin inhibitor aloxistatin (E64d) demonstrated robust anti-fibrotic effect by inhibiting patient-derived cardiac fibroblast proliferation, migration and extracellular matrix formation.
- Aloxistatin treatment reduced pro-inflammatory response in hiPSC-derived macrophages and rat living myocardial slices.
- Dual mode of action of aloxistatin underlines therapeutic potential to treat cardiac fibrosis and heart failure progression.

**Supplementary Information:**

The online version contains supplementary material available at 10.1007/s00109-026-02695-5.

## Introduction

Cardiovascular diseases (CVDs) remain the leading global cause of mortality, accounting for approximately 19.8 million deaths in 2022 [1]. A significant proportion of these cases are with ischemic cardiomyopathy (ICM) or non-ischemic cardiomyopathy, such as dilated cardiomyopathy (DCM). ICM typically develops following one or multiple myocardial infarctions resulting in scar formation within the injured area [2, 3]. In contrast, DCM is often idiopathic or genetic in origin and is characterized by diffuse interstitial fibrosis. Despite their different pathological backgrounds, both conditions involve chronic fibrotic remodeling of the heart, which irreversibly impairs the myocardial structure and function, ultimately reducing cardiac output [4, 5].

Cardiac fibrosis on cellular and molecular level is a dynamic, multi-stage process initiated by cellular stress or tissue injury, which triggers the release of proinflammatory cytokines. This leads to an inflammatory response characterized by the infiltration of immune cells such as macrophages and neutrophils, which secrete inflammatory mediators including interleukin-1β (IL-1β), interleukin-6 (IL-6), and transforming growth factor-β1 (TGFβ1). Resident cardiac fibroblasts subsequently differentiate into activated myofibroblasts marked by increased proliferative capacity, enhanced migratory activity and excessive production of extracellular matrix (ECM) components. The resulting myocardial stiffening impairs electrical conduction and diastolic or systolic function, resulting in progressive loss of cardiac performance [6–9].

Current FDA-approved pharmacological treatments for heart failure are based on the ESC guidelines [10] and primarily alleviate symptoms with compensatory function without halting the underlying fibrotic progression. To date, no therapy is yet approved that specifically targets cardiac fibrosis or tested agents have safety concerns, underscoring the urgent need for novel therapeutic strategies [11].

In this study, we focused on the members of the cysteine protease families, calpains and cathepsins, as potential therapeutic targets for cardiac fibrosis therapy. For example, calpain-1 (CAPN1) and calpain-2 (CAPN2) contribute to the activation of the inflammatory NFκB pathway and also participate in the pro-fibrotic signaling cascade [12–14]. In addition, the cathepsin family plays a critical role in the NLRP3 inflammasome activation and ECM remodeling [15, 16]. Both calpains and cathepsins are dysregulated during the progression of cardiac fibrosis [17–19] making them promising candidates for a drug repurposing strategy, an approach that identifies new therapeutic uses for existing drugs, thereby reducing development time, cost, and safety risks compared to de novo drug discovery [20].

Here, we suggest the use of aloxistatin as drug repurposing approach, a broad-spectrum cysteine protease inhibitor, as a potential therapeutic strategy to attenuate pro-fibrotic remodeling and preserve cardiac function. Aloxistatin was originally developed as a therapeutic agent for various forms of muscular dystrophy but did not achieve regulatory approval due to inconclusive clinical results [21]. Since the safety profile of aloxistatin is tolerable, it has been further explored in several drug repurposing studies for indications including neurodegenerative diseases, such as Alzheimer’s disease, where it inhibits calpain activity to preserve synaptic plasticity [22], as well as viral infections, including monkeypox and coronavirus, by reducing viral entry [23–25].

In this preclinical study, we evaluated anti-fibrotic potential of aloxistatin for the treatment of cardiac fibrosis applying human cardiac fibroblasts and macrophages *in vitro*, and *ex vivo* 3D rodent living myocardial slices combined with transcriptome profiling for mechanistic insight.

## Methods

### Cultivation of human cardiac fibroblasts

Human cardiac fibroblasts (HCF) derived from patients with either ischemic cardiomyopathy (Lot. 470Z011.5) or dilated cardiomyopathy (Lot. 452Z013.1, Lot. 436Z024.3) were purchased from PromoCell (Heidelberg, Germany). HCFs were cultivated as described before [26]. Cell concentration during splitting was determined by mixing 10 µL of suspension with equal volume of 0.4% trypan blue stain (Invitrogen, Darmstadt, Germany) and counting with Neubauer chamber.

### Treatment of HCF

If not indicated otherwise, HCFs were treated for 48 h with 100 µM Aloxistatin (S7393, Selleckchem, Munich, Germany) or DMSO control (A994.1, Roth) 24 h after seeding in cell culture medium. Pro-fibrotic stimulation was performed with 5 ng/mL TGFβ1 (130–095-067, Miltenyi Biotec, Bergisch Gladbach, Germany) or respective vehicle (veh) control (4 mM HCl containing 0.1% bovine serum albumin; 9048–46-8, Serva, Heidelberg, Germany).

### SiRNA transfection of HCFs

HCFs were transfected with 10 nM of either control siRNA-A (siCtrl) or siRNA targeting *CTSB* (siCTSB) or *CAPN2* (siCAPN2; all Santa Cruz Biotechnology, Dallas, TX, USA). SiRNAs were first formulated in Gibco™ Opti-MEM™ (31985070, Thermo Fisher Scientific, Waltham, MA, USA) with Lipofectamine RNAiMAX (13778150, Thermo Fisher Scientific) at a concentration of 4 µL per 1 mL transfection mix and incubated for 20 min at room temperature. Culture medium was aspirated and transfections were performed for 4 h at 37 °C and 5% CO_2_ in a humidified atmosphere. Afterwards, transfection mixes were removed and cells received culture medium with or without respective treatments.

### Cell migration

Cell migration activity of HCF was determined as described before [26]. Wound closure was documented every 2 h over a 24 h period with Gen5 by Bright Field High Contrast images and images in DAPI channel (${\lambda}_{Excitation}$= 377 nm; ${\lambda}_{Emission}$=447 nm) at Cytation 5 and BioSpa (Agilent, Waldbronn, Germany). Covered areas were calculated using Fiji Plugin [27].

### Cell proliferation assay

Prior seeding, the 96-well plate was coated with 0.1% galantine (G9391, Sigma Aldrich, Hamburg, Germany) at 37 °C for 30 min. HCF were seeded in a density of 5,000 cells per well and 24 h after start of drug treatment, 5-Bromo-2′-deoxyuridine (BrdU) labeling solution was diluted 1:100 in cell culture medium and 10 µL/well were added. After 24 h incubation, incorporated BrdU was detected by using Proliferation ELISA–BrdU kit (Roche, Mannheim, Germany) according to manufacturer’s instructions. Absorbance was detected at 370 nm (reference 490 nm) using the Cytation 5 (Agilent).

### Quantification of mRNA expression from cell culture

HCF were seeded at a concentration of 40,000 cells per well in a 24-well plate. After treatment of HCF, the medium as removed and HCF were harvested with 1 mL QIAzol Lysis Reagent (Qiagen, Hilder, Germany). RNA extraction was performed by adding 200 µL chloroform, vortexing for 15 s and incubating for 5 min at room temperature. Suspension was centrifuged at 12,000 × g and 4 °C for 5 min and 500 µL of aqueous phase was transferred to 500 µL isopropanol and 0.5 µL glycogen (10814–010, Thermo Fisher Scientific) was added. After 5 min incubation on ice, solution was centrifuged at 12,000 × g and 4 °C for 10 min, supernatant was discarded, pellet was washed with 75% ethanol and suspension was centrifuged for 10 min at 12,000 × g and 4 °C. Washing step was repeated once and pellet was dried at room temperature for 15 min. Pellet was dissolved in 15 µL RNase-free water (10977–035, Thermo Fisher Scientific) and incubated on ice for 3 min. Concentration and quantity was measured at Cytation 5 (Agilent) For gene expression analysis, complementary DNA (cDNA) was synthesized using the First Strand cDNA-Synthese Kit with oligo-dT Primers (K1612, Thermo Fisher Scientific) according to manufacturer’s instructions. Real-time quantitative polymerase chain reaction (RT-qPCR) followed using the Absolut Blue QPCR Mix, SYBR Green, low ROX (AB4322/B, Thermo Fisher Scientific) according to manufacturer’s protocol and with a final concentration of cDNA of 2 ng/µL and 0.5 µM of specific primer pair (Table 1). qPCR reaction was performed in 384-well plates using the Quant Studio 6 Flex system with QuantStudio™ Real-Time PCR software (ABI, Waltham, MA, United States).

**Table 1:** Human primer sequences

Gene name	forward	reverse
*ACTA2*	CCTGACTGAGCGTGGCTATT	GATGAAGGATGGCTGGAACA
*CDKN1A*	GCAGACCAGCATGACAGATTTC	GGATTAGGGCTTCCTCTTGGA
*COL12A1*	GAGCGCACACTGCCTATC	ACCACTAGCAGGATCCCACTT
*CTGF*	GTGTGCACCGCCAAAGAT	GTGTCTTCCAGTCGGTAAGC
*GUSB*	GACACCCACCACCTACATCG	CTTAAGTTGGCCCTGGGTCC
*CAPN1*	GGCATCTTCCATTTCCAGCTGT	ACACTAGCTTCCCGTCCTTG
*CAPN2*	GGCATACGCCAAGATCAACG	CAATGCCTCCGGTGAAGTCT
*CTSB*	ATGTGTGGGGACGGCTGTAA	GGATGGAGTACGGTCTGCAC
*CTSL*	TCCTGTGAAGAATCAGGGTCAG	GCCCAGAGCAGTCTACCAGA

### Enzyme-linked immunosorbent assay (ELISA)

HCFs were seeded in a density of 40,000 cells per well in a 24-well format and after treatment, supernatant was collected and centrifuged for 5 min at 300 × g and 4 °C. Secreted human matrix metalloprotease 2 (MMP2) and fibronectin 1 (FN1) levels were diluted 1:20 and 1:100, respectively, and quantified using DuoSet ELISA kits (DY902, DY1918-05, R&D Systems, Wiesbaden-Nordenstadt, Germany) following the manufacturer’s instructions.

### Quantification of reactive oxygen species

HCF were seeded with a density of 30,000 cells per well in a 96-well plate. After 24 h, treatment was added and maintained for 42 h. For DCFDA/H_2_DCFDA–Cellular ROS assay (Abcam, Cambridge, United Kingdom), cells were washed once with PBS and 50 µL per well 1 × assay buffer was added to the cells. After 45 min incubation at 37 °C, cells were washed with PBS once and respective treatments were added in 1 × assay buffer with or without 250 µM H_2_O_2_. Fluorescence intensity was measured at ${\lambda}_{Excitation}$= 485 nm and ${\lambda}_{Emission}$=535 nm for 2 h every 20 min using the Cytation 5 (Agilent).

### Animal experiments

Rat heart samples were obtained from Wistar rats, and the research related to animal’s use complied with all the relevant national regulations and institutional policies for the care and use of animals.

### Generation and cultivation of living myocardial slices

Living myocardial slices (LMS) from Wistar rat were generated as described in published protocols [28]. Briefly, left ventricular tissue was isolated and processed with a high precision vibratome (Model 7000SMZ2, Campden Instruments LTD, Leicestershire, United Kingdom) to obtain tissue sections with a thickness of approximately 300 µm. For modified 12-well plate or on-chip cultivation in IONOPTIX C-Pace (Westwood, USA), slices were trimmed into a 4 × 4 mm format and glued onto tissue holder using surgical histoacryl (1050052, B. Braun, Melsungen, Germany). Miniaturized rodent LMS were cultured in a 12-well plate format in M199 (P04-07050, Pan) with 3% penicillin/streptomycin (15140–122, Thermo Fisher Scientific) and 1:100 insulin-transferrin-selenium (P07-03210, PAN-Biotech, Aidenbach, Germany) at 37 °C and 5% CO_2_ and stimulated with the modified IONOPTIX C-Pace (pulse duration 5 ms, amplitude 6 V, frequency 0.2 Hz) as published before [29]. LMS were treated for 15 min, 4 h or 24 h with 100 µM aloxistatin or respective DMSO control.

### Quantification of RNA expression from *ex vivo* culture

LMS were harvested and transferred into 2 mL tubes (P000945-LYSKO-A.0, Precellys) with ceramic beads (91-pCS-CK 28P, Peglab) containing 1 mL QIAzol Lysis Reagent (Qiagen). Tissue was homogenized with Precellys Evolution (Bertin Technologies, Montigny-le-Bretonneux, France) by performing two cycles over 20 s with a speed of 5500 rpm and a pause of 5 s. RNA isolation was conducted as mentioned above. For gene expression analysis, cDNA was synthesized using the First Strand cDNA-Synthesis Kit with Random Hexamer Primers (K1612, Thermo Fisher Scientific) according to manufacturer’s instructions. RT-qPCR procedure was performed as mentioned before with the following primers (Table 2).

**Table 2:** Rat primer sequences

Gene name	forward	reverse
*18 s*	GAGAAACGGCTACCACATCCA	GCCTCGAAAGAGTCCTGTATTG
*Ctgf*	GAAGCAGAGTCGTCTCTGCA	AGAAAGCTCAAACTTGACAGGC
*Il1b*	TTGAGGCTGACAGACCCCAA	GCTTCTCCACAGCCACAATG
*Il6*	TCTCTCCGCAAGAGACTTCCA	ATACTGGTCTGTTGTGGGTGG
*Nppa*	TCTGATGGATTTCAAGAACCTGC	CAGAGAGGGAGCTAAGTGCC
*Nppb*	ATCCACGATGCAGAAGCTGCT	CCTTGGTCCTTTGAGAGCTGT
*Postn*	GTTCCTGTGTGACGTTGACC	CGGGGCAGCATTCATATAGC

### RNA sequencing

RNA for sequencing was extracted as described before. Aptitude of RIN values was assured using BioAnalyzer 2000 (Agilent) measurement. RNA samples were sequenced by Novogene using standard Illumina protocols (NovaSeq X Plus platform) for paired end 150 bp sequencing (30 M (HCF) or 40 M (LMS) reads per sample) after either poly(A) enrichment or rRNA depletion for HCF- and LMS-derived samples, respectively. After quality control of raw sequence reads with fastp [30], alignment to homo sapiens (GRCh38) or rattus norvegicus (GRCr8) reference genome for HCF and LMS datasets, respectively, was performed utilizing STAR [31]. Read counts per gene were determined with the STAR ‘GeneCounts’ quantification mode based on ENSEMBL human and rat transcript reference assembly version 113 and 115, respectively. All genes with at least 10 read counts in a number of samples less than the number of sequenced biological replicates per group were excluded from further analyses. Normalization of raw counts and subsequent calculation of differential gene expression was performed with DESeq2 package (version 1.46.0) [32] for R (version 4.4.1) with default parameters. All genes fulfilling adjusted p-value ≤ 0.05 and absolute log2 fold change (log2FC) ≥ 1 were deemed significantly differentially expressed. The gprofiler2 (version 0.2.3) package for R was used to identify over-represented terms from gene ontology, KEGG, WikiPathways, and Reactome databases in significantly differentially expressed genes (DEGs). All Venn diagrams were created using InteractiVenn webtool [33].

### Cultivation of human embryonic kidney cells

Human embryonic kidney (HEK) 293 FT cells were cultivated in T-75 flasks (90076, TPP, Sigma, Rödermakr, Germany) and received high-glucose Dulbecco’s modified Eagle’s medium (DMEM, 41965–039, Thermo Fisher Scientific), supplemented with 1% penicillin–streptomycin (PenStrep, 15140–122, Thermo Fisher Scientific) and 10% fetal bovine serum (FBS, 10437–028, Lot 2394347RP, Gibco) at 37 °C and 5% CO_2_.The medium was exchanged every two to three days and passaging was performed weekly. For splitting, HEK293FTs were rinsed with Dulbecco’s phosphate-buffered saline (PBS, 14190–094, Thermo Fisher Scientific) and incubated for 5 min at 37 °C with 0.05% trypsin–EDTA solution (25300–054, Thermo Fisher Scientific). After detachment of cells, cell culture medium was added, and suspension was centrifuged for 5 min at 4 °C and 300 × g. Cells were resuspended in culture medium and cell concentration was determined by mixing 10 µL of suspension with 10 µL of 0.4% trypan blue stain (Thermo Fisher Scientific) and counting with Neubauer chamber. For cultivation, 50,000 cells were seeded per flask.

### Nuclear factor (NF) κB reporter assay

HEK293FT were seeded in a density of 12,500 cells per well in a 48-well plate (92448, TPP, Sigma) and cultivated for 24 h. Cells were transfected with pSGN-luc plasmid expressing luciferase under NFκB promotor and pmir-Report β-Galactosidase plasmid expressing β-galactosidase for normalization [34]. For a final plasmid transfection of 100 ng per well, plasmids were diluted in OptiMEM (51985, Thermo Fisher Scientific) at a concentration of 1000 ng/mL. In parallel, Lipofectamine® 2000 (11668, Thermo Fisher Scientific) was diluted 1:200 in OptiMEM. After 5 min incubation at room temperature, both solutions were combined at equal volume and incubated for 20 min at room temperature. Medium of the cells was removed, and the transfection mix was added to the cells (200 µL per well). After 4 h at 37 °C, transfection mix was removed and medium containing 0.1% FBS and 1% PenStrep and compounds (Aloxistatin, S7393, Selleckchem; Loxistatin acid, S7392, Sellelckchem) or respective dimethyl sulfoxide (DMSO, A994.1, Roth) control. After 24 h of incubation under standard cultivation conditions, HEK293FT were incubated on ice for 10 min and cell suspension was centrifuged for 5 min at 4 °C and 300 × g. Supernatant was removed, pellets were lysed in 70 µL Cell Culture Lysis Reagent (E1500, Luciferase Assay System, Promega, Walldorf, Germany) on ice for 15 min and cleared by centrifuge at 8000 × g and 4 °C for 5 min. For luciferase detection, 5 µL of supernatant was diluted in 45 µL Cell Culture Lysis Reagent. After adding 50 µL Luciferase Assay Substrate (E1500, Luciferase Assay System, Promega) per well, luminescence was measured every 40 s over a 10 min period at Cytation 5 (Agilent). For β-galactosidase determination, 25 µL of supernatant was diluted with 25 µL Cell Culture Lysis Reagent (E1500, Luciferase Assay System, Promega) and 50 µL of Assay 2 × Buffer (E2000, β-Galactosidase Enzyme Assay System with Reporter Lysis Buffer, Promega) was added. After 30 min incubation at 37 °C, absorbance at 420 nm was detected at Cytation 5 (Agilent).

### Differentiation of human induced pluripotent stem cells (iPSCs) into macrophages

The differentiation of iPSC into iPSC-derived macrophages (iPSC-Mac) was performed as previously described [35]. In brief, the iPSC line CTi003-A (RRID: CVCL_C1W7) was cultivated in feeder-free monolayer culture on Geltrex™ (Gibco)-coated tissue culture plates under standard cell culture conditions. Cells were cultivated in Essential 8 (E8)-medium (Essential 6 (E6) medium (Dulbecco’s Modified Eagle Medium/Nutrient Mixture F-12 (DMEM/F-12; Gibco), 64 mg/L ascorbic acid 2-phosphate, 14 µg/L sodium selenite, 543 mg/L NaHCO_3_, 20 mg/L insulin, and 10.7 mg/L human recombinant transferrin (all from Sigma-Aldrich)), freshly supplemented with 100 ng/mL human basic fibroblast growth factor (hbFGF) and 2 ng/mL human transforming growth factor β 1 (hTGFb1) (both from PeproTech)), supplemented with 10 mM Y-27632 dihydrochloride (ROCK-Inhibitor (RI); Tocris). Passaging was performed every three days with Accutase® (Sigma-Aldrich). For differentiation, 5 × 10^5^ iPSCs per well were seeded in a CELLSTAR®−6-well plate (Greiner Bio-one) in E6-medium supplemented with 50 ng/mL hbFGF, 1 ng/mL hTGFb1, 50 ng/mL human vascular endothelial growth factor (hVEGF) and human bone morphogenetic protein 4 (hBMP4), 20 ng/mL human stem cell factor (hSCF) (all PeproTech) and 10 µM RI and placed on an orbital shaker (OMNI Life Science) at 70 rpm. On day 2, the medium was changed to E6-medium containing 50 ng/mL hVEGF and hBMP4, 20 ng/mL hSCF and 10 µM RI and the shakers rotation speed was increased to 85 rpm. On day 4 and 7, the cultivation medium was replaced to E6 medium supplemented with 50 ng/mL hVEGF and hBMP4, 20 ng/mL hSCF and 25 ng/mL human interleukin 3 (hIL-3; PeproTech). Macrophage production was initiated on day 10 by transferring the aggregated myeloid cell forming complexes onto an adherent 6-well tissue culture plate (TPP) and medium was changed to X-VIVO® 15 (Lonza) containing 2 U/mL penicillin–streptomycin, 2 mM L-glutamine, 50 µM β-mercaptoethanol (all Gibco) supplemented with 25 ng/mL hIL-3 and 50 ng/mL human macrophage colony stimulating factor (hM-CSF, PeproTech). Fresh macrophages were harvested during each medium change by filtering through a 70 µm gaze (Sarstedt), which was done once or twice per week.

### Evaluating anti-inflammatory drug response in challenged hiPSC-Macs

To assess the anti-inflammatory property towards a pro-inflammatory stimulus on macrophages, 5 × 10^5^ iPSC-Mac were pre-seeded in 12-well for 3 days in X-VIVO 15®, 2 U/mL Penicillin–Streptomycin, 2 mM L-Glutamin, 50 µM β -mercaptoethanol supplemented with 50 ng/mL hM-CSF. Cells were then co-treated with 5 ng/ml human interferon γ (hIFNγ; PeproTech) and 100 µM aloxistatin or respective DMSO control (Roth). After 24 h, cells were collected and stained for flow cytometry. Therefore, cells were incubated in PBS containing FcR Blocking Reagent (1:50; Miltenyi Biotec, 130059–901) and Zombie Aqua™ Fixable Viability Kit (1:250; Biolegend #423101) for 10 min at room temperature. Thereafter, cells were stained with HLA-DR-APC (1:100; Biolegend, 307610) in FACS buffer (PBS, 2 mM Ethylenediaminetetraacetic acid ((EDTA); Carl Roth) and 2% FBS) for 20 min at 4 °C in the dark. Samples were examined using a CytoFLEX S (Beckman Coulter) and data were analyzed with FlowJo software v10.10.0 (BD Bioscience).

### Statistical analyses

All bar graphs with statistical analyses for cell culture and living myocardial slices experiments were prepared using GraphPad Prism 10. Normal distribution of data was assessed with Shapiro–Wilk test and statistical analyses were determined with either unpaired t-test, two-way, or three-way analysis of variance (ANOVA) and *post-hoc* pairwise comparisons corrected according to Dunnett’s or Sidak’s method. Graphs represent mean values ± standard deviation. *P*-values ≤ 0.05 were regarded as statistically significant.

## Results

### Aloxistatin reduces migration and proliferation rate of diseased HCFs

Human cardiac fibroblasts (HCFs) are central effector cells in the development and progression of myocardial fibrosis, where their excessive proliferation, migration and extracellular matrix deposition drives scar formation progressively impairing cardiac performance [5]. Limiting the excessive wound-healing response of HCF therefore represents a key therapeutic goal.

**Fig. 1:**
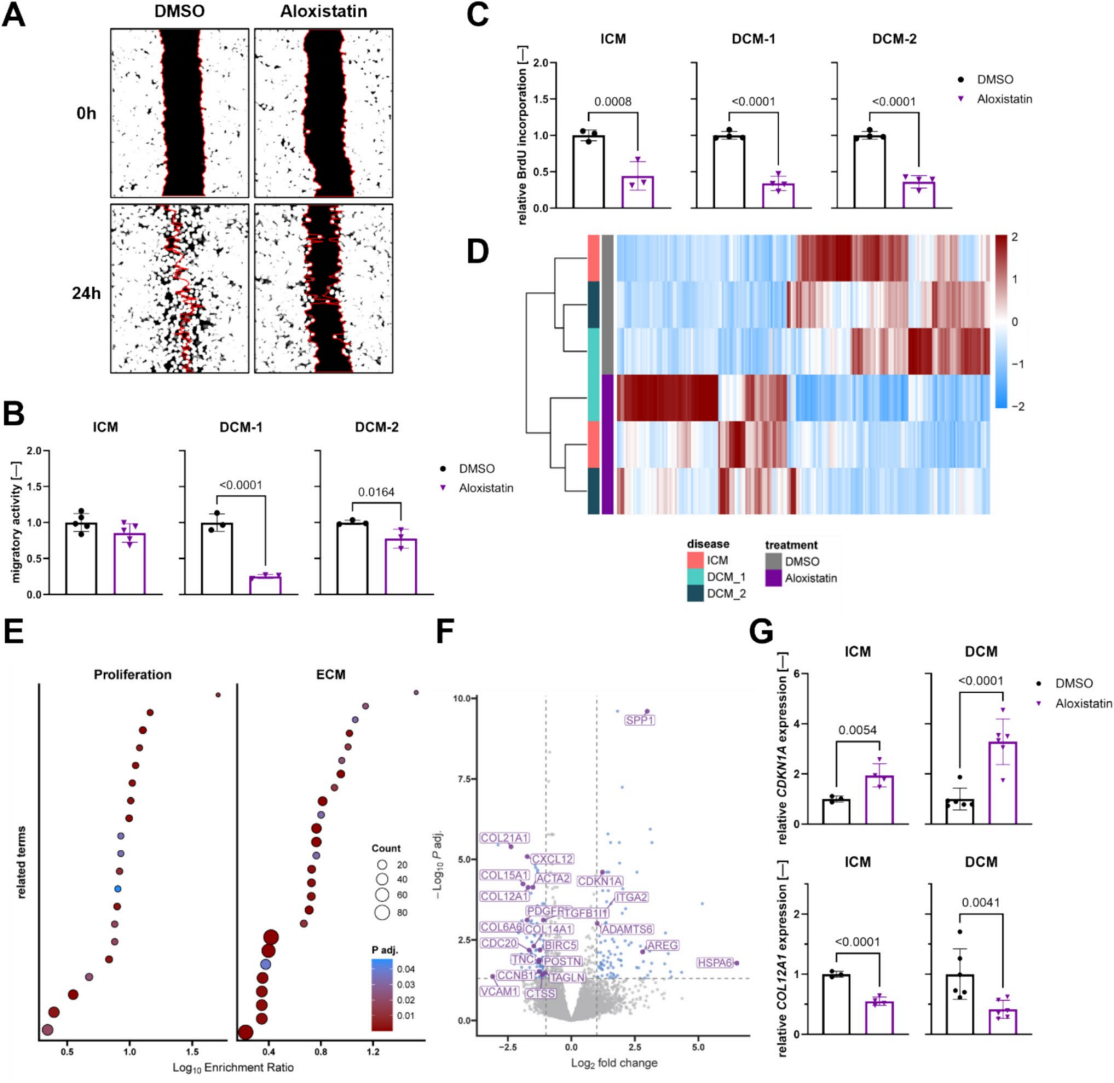
Aloxistatin treatment on diseased human cardiac fibroblasts impacts their migration and proliferation capacity. **A** Representative images of wound healing assay analysis of aloxistatin-treated human cardiac fibroblasts (HCF) with ischemic cardiomyopathy (ICM) and dilated cardiomyopathy (DCM) background and **B** respective quantification for migratory activity of relative wound areas covered over time (*n* = 3). **C** Relative cell proliferation of HCF with different pathological backgrounds after aloxistatin treatment measured over 24 h cultivation period (*n* = 3–4). **D** Heatmap of differentially expressed genes (DEGs; adjusted *p*-value ≤ 0.05, |log_2_ fold change|≥ 1) between DMSO control and aloxistatin-treated HCFs. **E** Analysis of DEGs for overrepresented terms from GO, KEGG, Reactome, and WikiPathways databases related to proliferation and extracellular matrix (ECM). **F** Volcano plot highlighting regulation of gene expression comparing aloxistatin and DMSO groups. Annotated candidates are related to ECM, cell cycle or fibrosis aspects. **G** Relative mRNA expression of cyclin-depended kinase inhibitor 1 A (*CDKN1A*) and collagen type 12a1 (*COL12A1*) after aloxistatin treatment (*n* = 3–6). DMSO, dimethyl sulfoxide; ICM, ischemic cardiomyopathy; DCM, dilated cardiomyopathy

To explore the antifibrotic potential of the cathepsin and calpain inhibitor aloxistatin, its effects on the biology of patient-derived human cardiac fibroblasts (HCFs) with ischemic cardiomyopathy (ICM) or dilated cardiomyopathy (DCM) backgrounds were assessed. Aloxistatin treatment significantly reduced migratory activity in DCM-derived HCFs but not in HCFs with ICM background (Fig. 1A, B). Furthermore, decreased proliferation in both ICM- and DCM-derived HCFs could be observed in response to aloxistatin treatment (Fig. 1C). However, no influence on general cellular viability was observed (Supplemental Fig. 1 A). We furthermore screened for potential cardio- and hepatotoxic side effects, two of the most common causes of post-marketing withdrawal of therapeutics [36], applying porcine living myocardial slices and human precision-cut liver slices, respectively. Advocating for the safety of aloxistatin, no impact on cardiac electrophysiology was visible in effective refractory period measurements using dual-pacing strategy (Supplemental Fig. 1B) [37]. Additionally, liver slices remained viable over 24 h after exposure to escalating concentrations of aloxistatin as measured in WST-1 and LDH assays (Supplemental Fig. 1 C).

### Transcriptomic profiling indicates regulation of cell proliferation and ECM formation after aloxistatin treatment

To validate the impact of aloxistatin on HCF functionality, we investigated mRNA expression profiles upon Aloxistatin treatment in comparison to solvent control (DMSO) in ICM and DCM-derived HCFs. Sequencing of mRNA revealed 197 differentially regulated genes after aloxistatin treatment in the dataset (Fig. 1D, Supplemental Fig. 1D). Overrepresentation analyses of aloxistatin in these DEGs validated previous findings from BrdU assays with multiple proliferation-related terms such as condensation of prophase chromosomes, G2/M DNA damage checkpoint, and regulation of cell population regulation, found among significantly enriched pathways. Additionally, aloxistatin showed a strong influence on ECM-related processes, e.g. extracellular matrix organization, collagen formation, assembly of collagen fibrils and other multimeric structures (Fig. 1E). In particular, aloxistatin treatment downregulated several collagen types such as *COL12A1* and *COL21A1* and fibrosis-associated markers and deregulated cell cycle regulation-related markers such as cyclin-dependent kinase inhibitor 1 A (*CDKN1A*) and surviving (*BIRC5*) (Fig. 1F). In line, qPCR-based gene expression analysis of HCFs validated the deregulation of *CDKN1A* and collagen type 12A1 (*COL12A1*) by aloxistatin highlighting its effect on proliferation and ECM formation independent of the cardiomyopathy background (Fig. 1G).

### TGFβ1–induced fibroblast activation and ECM remodeling in HCFs is inhibited by aloxistatin treatment

**Fig. 2:**
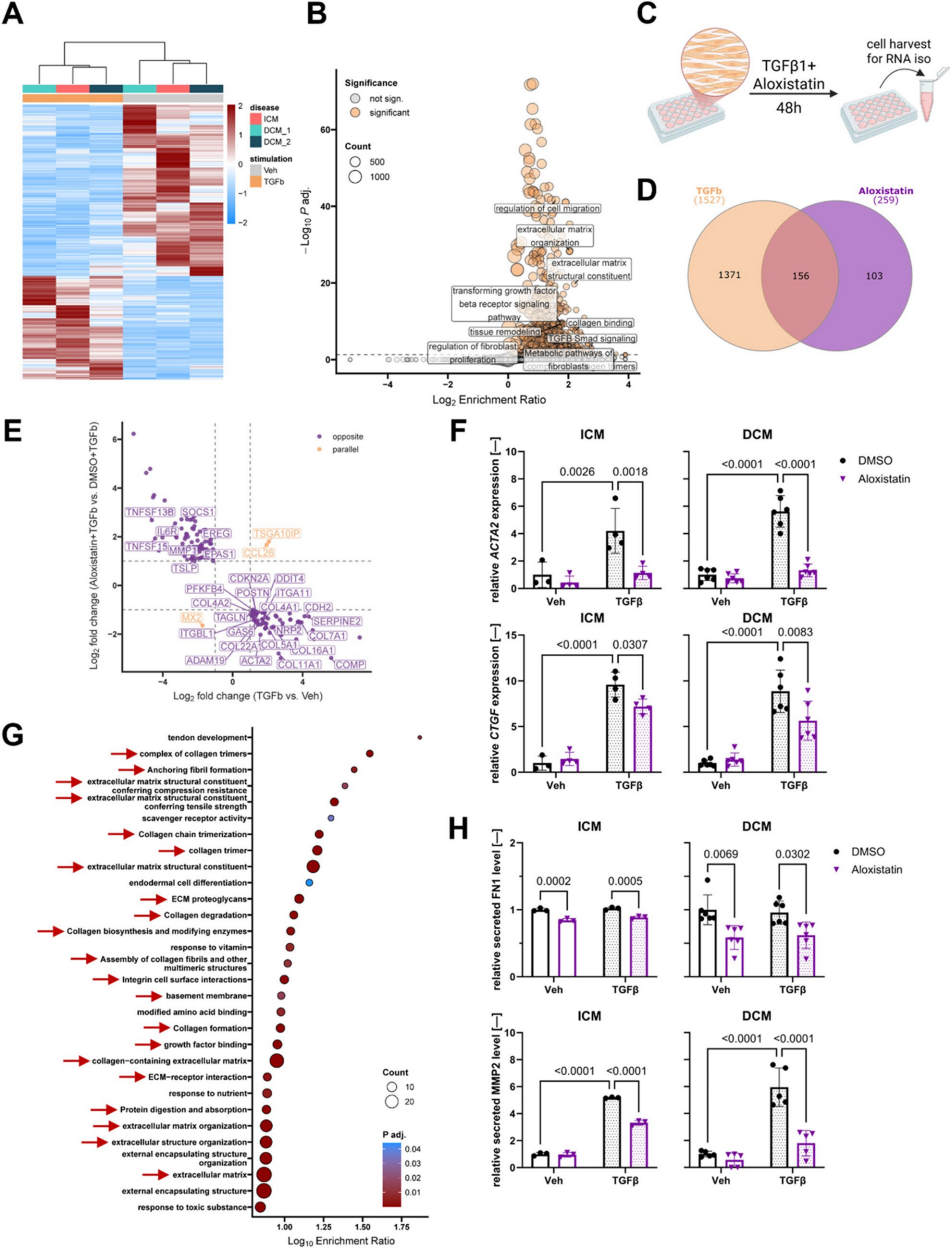
Aloxistatin attenuates TGFβ1–induced fibroblast activation and ECM remodeling. **A** Heatmap of differentially expressed genes (DEGs; adjusted *p*-value ≤ 0.05, |log_2_ fold change|≥ 1) between vehicle (veh) control and transforming growth factor β1 (TGFβ1)-treated HCFs with ICM and DCM backgrounds. **B** Overrepresentation analysis in DEGs in HCFs after TGFβ1 stimulation using GO, KEGG, Reactome, and WikiPathways databases as references. Selected fibrosis-associated terms are highlighted. **C** Schematic illustration of experimental design for investigating the effect of aloxistatin on TGFβ1-stimulated HCFs. HCFs were simultaneously stimulated with TGFβ1 and treated with aloxistatin. After 48 h, HCFs were collected for RNA isolation. **D** Overlap of deregulated genes in HCF after TGFβ1 stimulation and aloxistatin treatment. Values represent the number of genes in each category. **E** Fold changes in expression of overlapping genes from **D** in TGFβ1-stimulated HCFs (x-axis) and aloxistatin-treated TGFβ1-stimulated HCFs (y-axis) compared to respective controls. **F** Relative mRNA expression of fibrosis-associated markers α-smooth muscle actin 2 (*ACTA2*) and connective tissue growth factor (*CTGF*) after TGFβ1 stimulation and aloxistatin treatment (*n* = 3). **G** Top 30 terms overrepresented in opposite DEGs from **E** aloxistatin with GO, KEGG, Reactome, and WikiPathways reference databases. **H** Relative protein level of secreted fibronectin 1 (FN1) and matrix metalloproteinase 2 (MMP2) of TGFβ1-stimulated and aloxistatin treated HCFs. DMSO, dimethyl sulfoxide; ICM, ischemic cardiomyopathy; DCM, dilated cardiomyopathy

ICM and DCM are progressive disorders that ultimately result in end-stage heart failure [2, 3]. To further drive these chronically diseased HCFs toward an acutely activated phenotype, TGFβ1 was applied as pro-fibrotic stimulation and respective vehicle (veh) control. mRNA sequencing revealed a total of 1527 differentially expressed protein-coding genes upon TGFβ1 stimulation in HCFs (Fig. 2A). In addition, a multitude of fibrosis-related terms were significantly overrepresented among these DEGs (Fig. 2B).

To assess whether the anti-fibrotic activity of aloxistatin persists under pro-fibrotic conditions, HCFs were stimulated with TGFβ1, treated with aloxistatin and analyzed by mRNA sequencing (Fig. 2C). The previously identified 1527 deregulated candidates after TGFβ1 stimulation were compared to the 259 significantly regulated protein coding genes after TGFβ1 stimulation and aloxistatin treatment resulting in an overlap of 156 common DEGs (Fig. 2D). Notably, aloxistatin almost exclusively oppositely regulated expression of overlapping candidates compared to TGFβ1 stimulation (Fig. 2E). Of great importance, opposite regulation of key pro-fibrotic marker genes α-smooth muscle actin (*ACTA2*) and connective tissue growth factor (*CTGF*) by aloxistatin in TGFβ1-stimulated HCFs derived from both ICM and DCM patients could be validated using qPCR (Fig. 2F). To gain a more comprehensive picture of molecular processes that aloxistatin counteracts in the acute pro-fibrotic HCF model, we subjected the oppositely regulated genes to overrepresentation analysis. Importantly, ECM-related terms appeared with high frequency among top 30 enriched pathways (Fig. 2G). By further exploring these findings on protein level, application of aloxistatin on ICM- and DCM-derived HCFs resulted in a downregulation of secreted fibronectin 1 (FN1) while TGFβ1 stimulation had no effect on FN1 secretion (Fig. 2H). Of great interest for functional outcome, aloxistatin effectively attenuated the prominent upregulation of matrix metalloprotease 2 (MMP2) secretion upon TGFβ1 exposure (Fig. 2H).

### Preclinical modeling of early profibrotic signatures in living myocardial slices

**Fig. 3:**
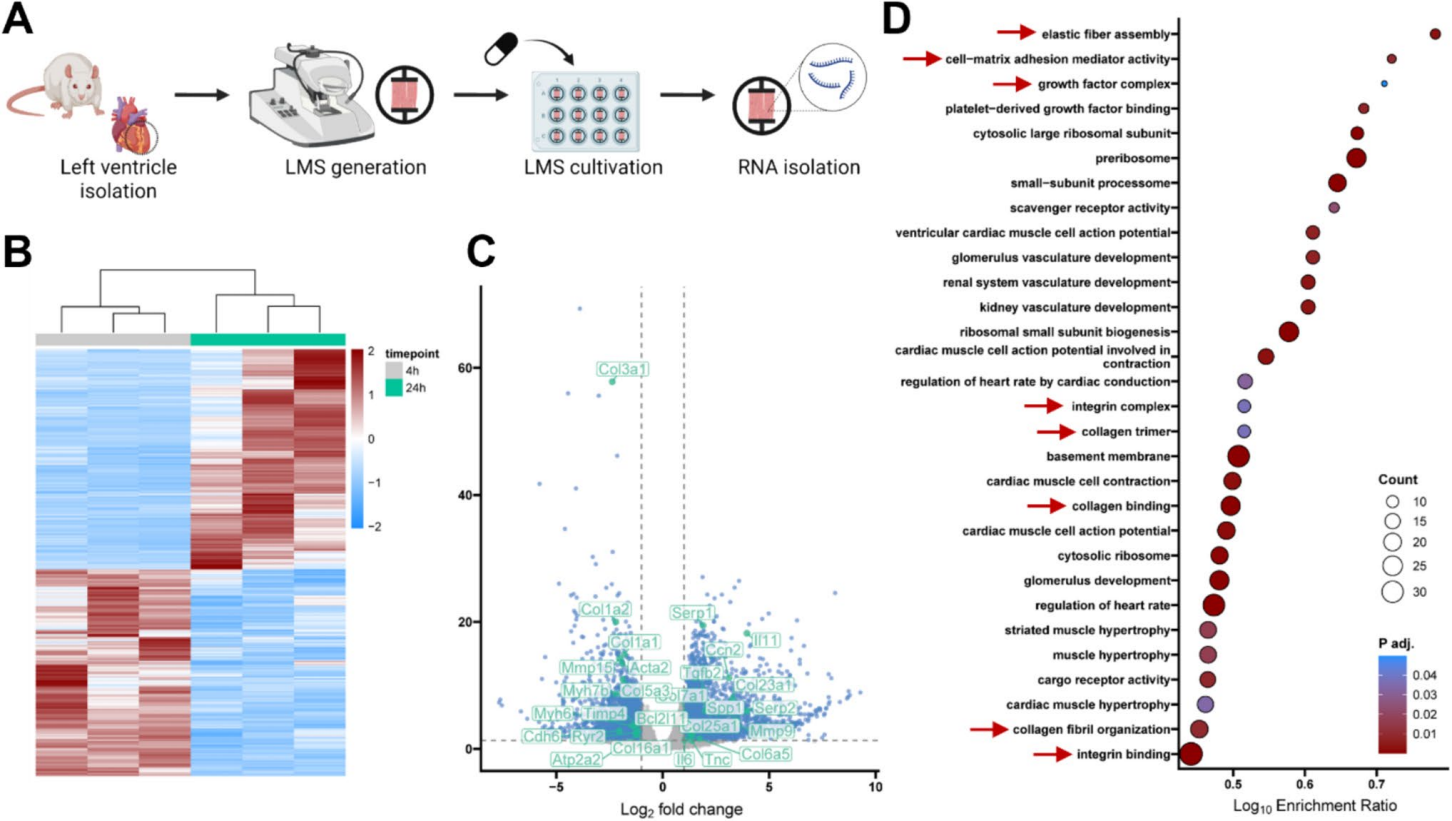
Living myocardial slices develop profibrotic gene expression program over time. **A** Schematic representation of experimental setup for measuring gene expression regulation in living myocardial slices (LMS). **B** Heatmap of differentially expressed genes (DEGs; adjusted *p*-value ≤ 0.05, |log_2_ fold change|≥ 1) between LMS cultivated for 4 h and 24 h. **C** Volcano plot highlighting regulation of gene expression comparing cultivation of LMS for 4 h and for 24 h. Labelled candidates are mainly related to extracellular matrix, fibrosis or inflammation aspects. **D** Overrepresentation analysis in DEGs from **B**, **C** with ECM-related terms highlighted by red arrows. Top 30 terms (GO, KEGG, Reactome, WikiPathways) are displayed. LMS experiments were performed with *n* = 3

To bridge previous experimental findings towards clinical impact and translation, we utilized the *ex vivo* model of miniaturized 3D biomimetic living myocardial slices (LMS), a highly predictive platform reflecting key characteristics of cardiac biology *in vivo* (Fig. 3A). This 3D tissue system maintains the native cardiac architecture, extracellular matrix organization, and cell–cell interactions, thereby allowing the study of aloxistatin’s potential anti-fibrotic effect in a structurally intact environment [38]. An initial approach comparable to our *in vitro* experimental setup utilizing Tgfβ1 to induce a pro-fibrotic phenotype in LMS did not result in changes of pro-fibrotic marker gene expression (Supplemental Fig. 2 A). Considering the stress exerted on the tissue during the process of LMS preparation and subsequent culture, we hypothesized that possible Tgfβ1-related pro-fibrotic signals could be already saturated. We therefore analyzed expression of aforementioned markers in LMS cultivated for 15 min, 4 h and 24 h. Interestingly, upregulated expression of interleukin 1β (*Il1*β), interleukin 6 (*Il6*), periostin (*Postn*) and *Ctgf* hinted at pro-inflammatory and pro-fibrotic events occurring in LMS within this early time frame of culture. Additional upregulation of cardiac stress and hypertrophy markers atrial natriuretic peptide (*Nppa*) and B-type natriuretic peptide (*Nppb*) over time insinuate that plain cultivation of LMS might be sufficient to model adverse myocardial remodeling (Supplemental Fig. 2B). To confirm this assumption, we performed bulk RNA sequencing of rat LMS and compared 24 h with 4 h cultivation timepoints. In total, 5355 significant deregulated genes were found in this analysis with hierarchical clustering and principal component analysis clearly separating 4 h and 24 h timepoints (Fig. 3B, Supplemental Fig. 2 C). Differentially expressed genes over time contain various candidates associated with ECM remodeling and inflammation (Fig. 3C) which is compellingly reflected in overrepresented pathways/processes (Fig. 3D).

### Aloxistatin therapy halts inflammatory response in rat LMS

**Fig. 4:**
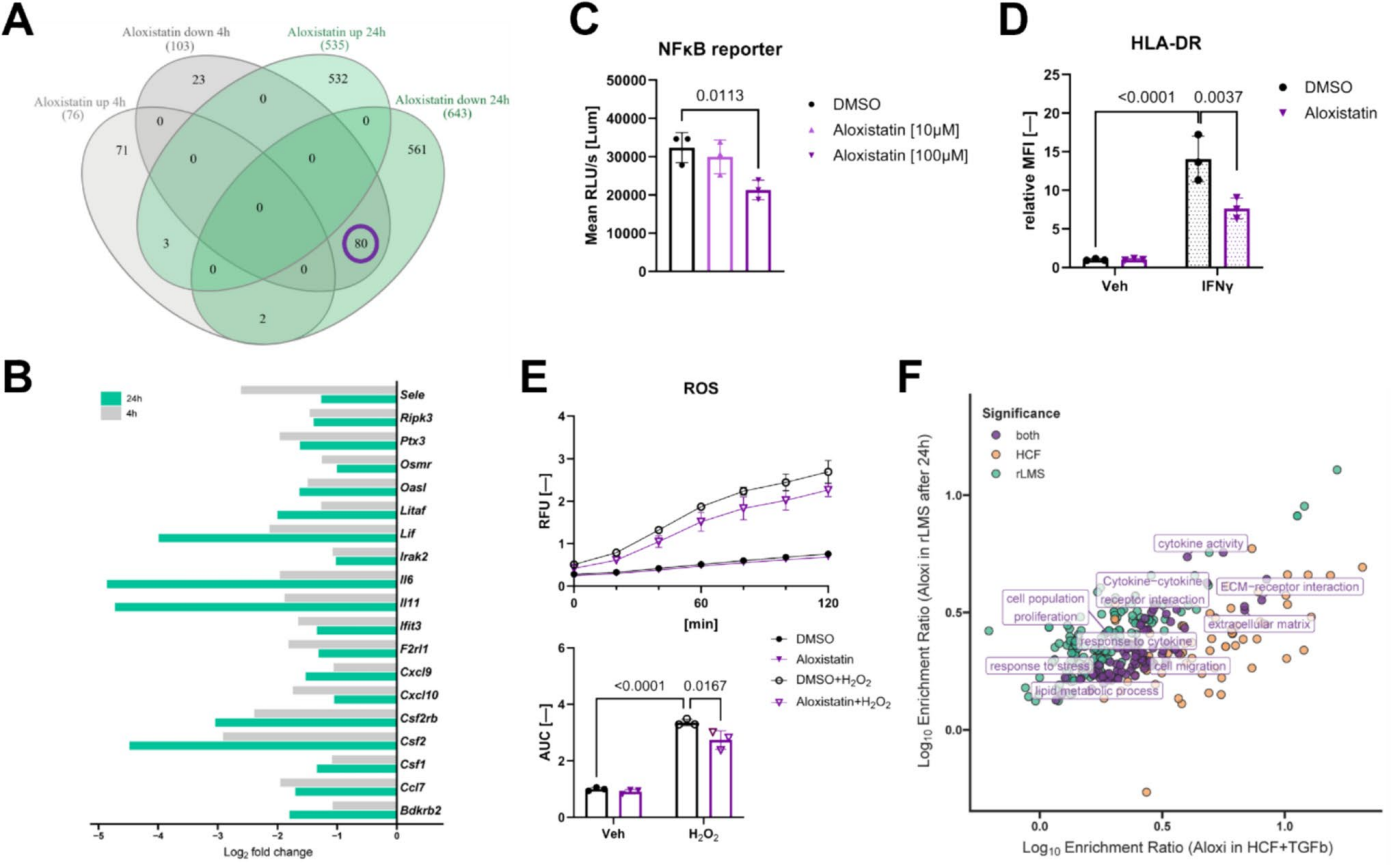
Aloxistatin inhibits inflammatory signaling and lowers oxidative stress. **A** Venn-diagram showing the overlap of regulated genes in RNA sequencing of rat living myocardial slices (LMS) treated with aloxistatin for 4 h and 24 h compared to respective dimethyl sulfoxide (DMSO) control. **B** Fold change of inflammation-related candidates significantly downregulated by aloxistatin treatment at both investigated timepoints. **C** NFκB signaling reported by luciferase activity in human embryonic kidney cells after 24 h treatment with aloxistatin [10 µM, 100 µM] (*n* = 3). **D** Human leukocyte antigen-DR isotype (HLA-DR) expression in interferon γ (IFNγ) [5 µg/mL] stimulated or/and aloxistatin-treated induced pluripotent stem cell-derived macrophages 24 h after treatment (*n* = 3). **E** Reactive oxygen species (ROS) levels assessed by monitoring fluorescence intensity in DCFDA-stained HCF over time after H_2_O_2_ stimulation or/and aloxistatin treatment (top). Area under curve (AUC) analysis of ROS levels over time (bottom; *n* = 3). **F** Comparison of significant term enrichment (references: GO, KEGG, Reactome, WikiPathways) in overrepresentation analyses from differentially expressed genes in TGFβ1-stimulated HCFs after aloxistatin-treatment (x axis) and LMS cultivated for 24 h under aloxistatin exposure (y axis). RFU, relative fluorescence units

*Ex vivo*, rat LMS were treated with aloxistatin for 4 h to examine early responses and for 24 h to evaluate effects beyond the early acute phase. After 4 h aloxistatin treatment, 179 genes were significantly deregulated in response to aloxistatin compared to DMSO control group while exposure for 24 h led to significantly regulation of 1178 genes. Interestingly, the majority of the 103 downregulated genes after 4 h of aloxistatin treatment were also found to be downregulated after 24 h (Fig. 4A). Among those, many inflammation-associated candidates such as selectin E (*Sele*), leukemia inhibitory factor (*Lif*), interleukin 6 (*Il6*), interleukin 11 (*Il11*) and colony stimulating factor 2 (*Csf2*) were either consistently or incrementally downregulated over time by aloxistatin treatment (Fig. 4B).

To examine the anti-inflammatory effects of aloxistatin in more mechanistic detail, we first assessed NFκB activity, a hallmark of the inflammatory signaling pathway [15]. Aloxistatin treatment resulted in a significant reduction of NFκB activity in HEK293FT reporter cells (Fig. 4C). To further validate these findings in a translational immune-relevant model, human induced pluripotent stem cell (iPSC)-derived macrophages were polarized into the pro-inflammatory phenotype using interferon-γ (IFNγ), which markedly increased the expression of the activation marker HLA-DR [39]. Aloxistatin treatment attenuated this pro-inflammatory response by repressing IFNγ-induced HLA-DR expression (Fig. 4D). Since inflammatory activation is often accompanied by increased oxidative stress, we next investigated whether aloxistatin modulates reactive oxygen species (ROS) production in HCFs, as ROS serve as mediators linking inflammatory signaling to fibrotic remodeling through the activation of redox-related pathways [40]. H_2_O_2_-challenged HCFs revealed a lowered ROS production upon aloxistatin treatment (Fig. 4E).

Beforementioned investigations on *in vitro* HCFs as master regulators of fibrosis and native tissue-derived *ex vivo* LMS validated the effect of aloxistatin in the cardiac fibrosis context. Of utmost importance, analyzing the enrichment terms of significant deregulated genes following aloxistatin application of TGFβ-stimulated HCFs and 24 h cultured LMS revealed that aloxistatin modulates key biological processes involved in fibrotic progression across both models. The overlapping pathways were primarily related to inflammatory response such as cytokine activity or response to cytokine and related to fibrotic remodeling such as cell population proliferation, cell migration and ECM-receptor interaction (Fig. 4F).

### Anti-fibrotic effects of aloxistatin are partially mediated by CAPN2 inhibition

**Fig. 5:**
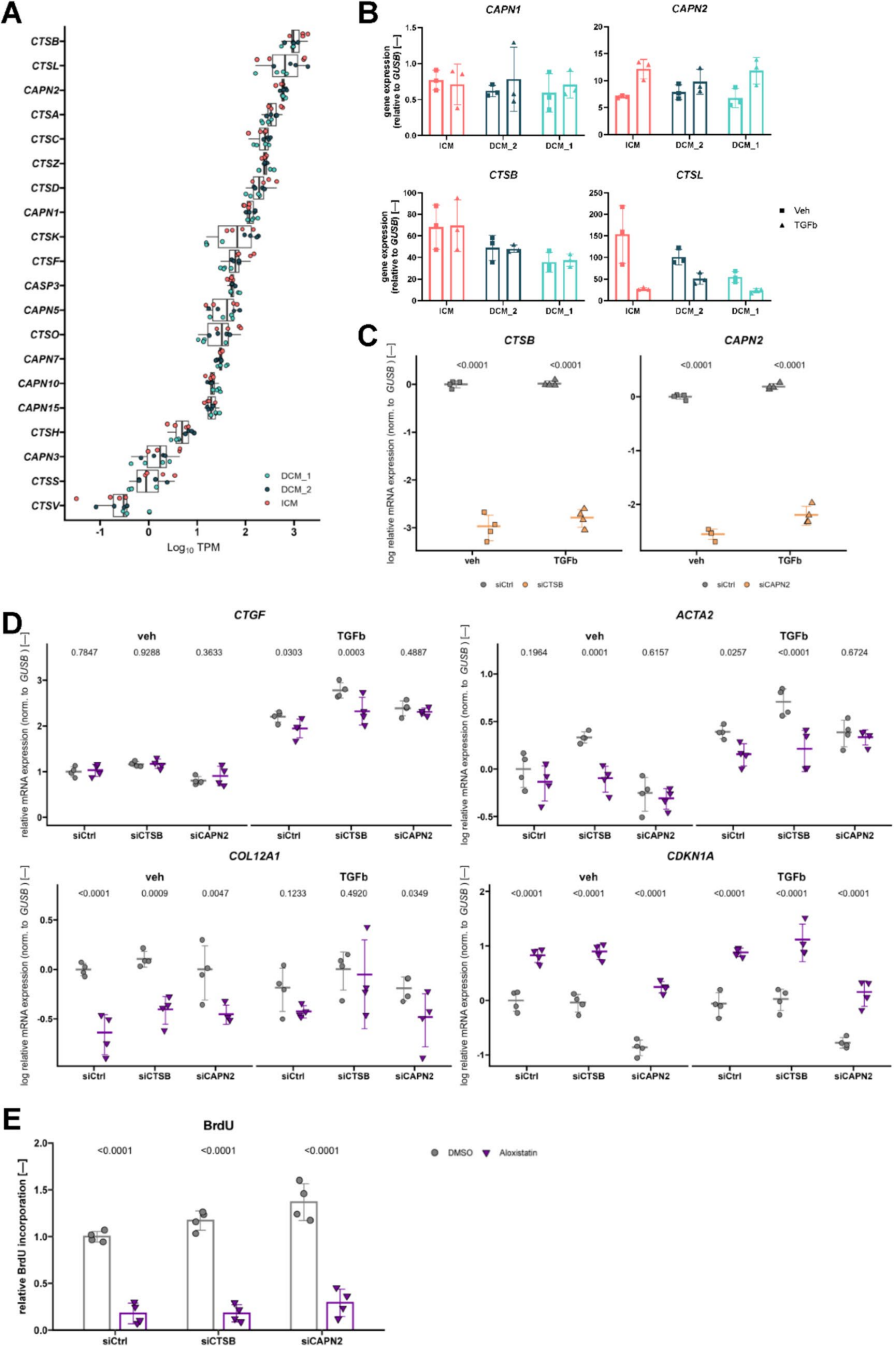
*CAPN2* is a key mediator of aloxistatin effects on HCFs. **A** Transcript per million (TPM) values of cathepsin and calpain family members in bulk mRNA sequencing of HCFs. **B** Expression levels of indicated targets in HCFs stimulated with or without 5 ng/mL transforming growth factor β1 (TGFb) for 48 h assessed via qPCR (*n* = 3). **C** mRNA expression of *CTSB* (left) and *CAPN2* (right) in ischemic cardiomyopathy (ICM)-derived HCFs after transfection of 10 nM siRNAs for 4 h and stimulation as depicted (*n* = 4). **D** Pro-fibrotic marker gene expression measured with qPCR in ICM-derived HCFs transfected with 10 nM siRNA as outlined for 4 h, stimulated with 5 ng/mL TGFb or vehicle control, and treated with 100 µM aloxistatin or dimethyl sulfoxide (DMSO) control for 48 h (*n* = 4). **E** Proliferation rates of ICM-derived HCFs transfected with either control (siCtrl), *CTSB*- (siCTSB), or *CAPN2*-targeting (siCAPN2) siRNA and exposed to 100 µM aloxistatin or respective DMSO control determined via incorporation of 5-bromo-2'-deoxyuridine into replicated DNA over 24 h (*n* = 4)

Finally, we sought to identify key molecular interactions that underlie the previously discovered anti-fibrotic effects of aloxistatin. As an unspecific inhibitor of calpains and cathepsins, we first interrogated our HCF bulk RNA-seq data for expression strength to narrow down the list of putative key mediators (Fig. 5A). We found *CTSB* and *CTSL* as well as *CAPN2* and *CAPN1* to be the most abundantly expressed members of the cathepsin and calpain family, respectively. We validated the expression of those candidates in all HCF lots utilized in this study using qPCR (Fig. 5B). Upon TGFβ1 stimulation, *CAPN2* expression tended to be slightly elevated while *CAPN1* and *CTSB* showed no deviation. Conversely, *CTSL* mRNA abundancy was repressed by TGFβ1 treatment and, interestingly, showed high inter-donor variability. We therefore elected to further investigate *CTSB* and *CAPN2* as potential mediators of the impact of aloxistatin on fibroblast biology. Successful knockdown (KD) of *CTSB* and *CAPN2* was observed in HCFs upon transfection with respective siRNAs and was preserved after stimulation with TGFβ1 (Fig. 5C). We next investigated whether KD of *CTSB* or *CAPN2* influences the effect of aloxistatin on pro-fibrotic gene expression (Fig. 5D). Interestingly, *CTSB* KD tended to enhance pro-fibrotic TGFβ1-effects which was rescued by aloxistatin. Of note, KD of *CAPN2* abrogated the inhibitory effect of aloxistatin on fibroblast activation markers *CTGF* and *ACTA2* under TGFβ1 stimulation. However, in terms of *COL12A1* and *CDKN1A* we could not find such effects. Depletion of CAPN2 significantly decreased expression of cell cycle inhibitor *CDKN1A* which was elevated again by aloxistatin application. This was also reflected on a functional level in BrdU assay (Fig. 5E).

## Discussion

To date, no existing therapeutic approach directly acts on reverting cardiac fibrosis. Clinical blockbuster drugs from gliflozin family or incretin mimetics support cardiac healing primarily via extra-cardiac routes [41–43]. Herein, aloxistatin demonstrated high potential as a drug repurposing candidate for the treatment of cardiac fibrosis both at early and late stage. Aloxistatin treatment showed both anti-inflammatory and anti-fibrotic impact in HCFs, LMS, and macrophages, highlighting its relevant impact on key cellular processes driving fibrosis progression as an engine of heart failure. The recent approval of nerandomilast, a selective inhibitor of PDE4 reducing the expression of inflammatory cytokines and pro-fibrotic growth factors, for the treatment of idiopathic pulmonary fibrosis (IPF) underlines the importance of targeting these two axes in fibrotic diseases [44, 45].

The anti-inflammatory effect of cysteine protease inhibitor aloxistatin, particularly relevant to the early stages of fibrosis, could be shown in 2D cell culture conditions as well as 3D rat LMS. The molecular targets of aloxistatin play crucial roles in regulating the canonical NFκB signaling pathway and inflammasome activation. The endogenous inhibitor IκBα is a known substrate of CAPN1 and CAPN2 and calpain-mediated cleavage of IκBα leads to its degradation, thereby facilitating NFκB activation and nuclear translocation [12]. Once activated, NFκB promotes the transcription of pro-inflammatory and pro-fibrotic mediators including *IL1*β, *IL18*, *TGF*β*1*, *NLRP3* and caspase-1. In parallel, the lysosomal cathepsin B (CTSB) and cathepsin L (CSTL), can escape into the cytosol through lysosomal leakage under cellular stress or heart failure conditions [46], where it contributes to caspase 1 activation and NLRP3 inflammasome assembly [15, 47]. Beyond that, lysosomal membrane damage might be additionally initiated by excessive ROS production within the cell leading to increase of NLRP3 inflammasome activation with subsequent cleavage of pro-IL1β and pro-IL18 into their active and secreted forms. These cytokines, primarily released by macrophages, amplify the inflammatory response and promote crosstalk with other cardiac cell types [40, 48]. In line with cathepsin inhibition by aloxistatin, siRNA knockdown of CTSB reduced caspase-1 activation and IL1b secretion in murine vascular endothelial cells [49]. Thus, aloxistatin’s interference with CAPN1, CAPN2, CTSB, CTSL and ROS reduction potentially underlies its ability to reduce NFκB-driven inflammatory signaling and inflammasome activation, contributing to its overall anti-inflammatory effect. To proof whether aloxistatin’s anti-inflammatory action extend beyond 2D cell culture conditions, we next applied 3D rat LMS, an *ex vivo* model that retains the native multicellular architecture, extracellular matrix composition, and mechanical environment of cardiac tissue [38]. LMS treated with aloxistatin validated anti-inflammatory effect by downregulation of various inflammatory markers, e.g. *Il6* and *Il11* [50, 51], on transcriptional level.

In further developing heart failure, the inflammatory cascade is followed by progressive pro-fibrotic remodeling and stiffening of cardiac tissue are mainly related by the activation of cardiac fibroblasts, which exhibit excessive ECM synthesis, enhanced migration, and proliferation [6, 52]. Aloxistatin treatment on ICM- and DCM-derived HCFs reduced their proliferation capacity, inhibited migratory activity and suppressed collagen expression confirming its anti-fibrotic effect on pathological fibroblast biology. When aloxistatin was applied to TGFβ1-stimulated fibroblasts, it significantly repressed the expression of acute fibrosis markers, including *ACTA2* and *CTGF*, and downregulated multiple ECM components including various collagen isoforms and *POSTN*, which are essential for collagen formation and matrix remodeling [52]. Under physiological conditions, calpain activity is tightly controlled by its endogenous inhibitor calpastatin. In fibrotic diseases, however, the calpain/calpastatin system is often dysregulated resulting in maladaptive hypertrophy and HF [18]. Our KD experiments showed that *CAPN2* depletion abrogated the effect of aloxistatin on fibroblast activation marker expression, however, had no impact regarding fibroblast proliferation inhibition and ECM marker repression. This insinuates that some of the observed anti-fibrotic effects of aloxistatin might me mediated through normalization of the calpain system. Nevertheless, further analyses are warranted to generate a more comprehensive understanding of the underlying mechanisms of aloxistatin’s impact on fibroblasts.

Thus, targeting this imbalance with aloxistatin represents a promising approach to restore proteolytic homeostasis. In this context, preclinical studies have demonstrated that pharmacological calpain inhibition with SNJ-1945 attenuates post-myocardial infarction remodeling in rats [53]. However, the herein gathered results from bulk RNA-sequencing analyses and functional assays in different 2D and 3D test systems cannot conclusively explain the mechanism underlying the anti-inflammatory and anti-fibrotic effects of aloxistatin. For example, resolving the single cell impact of aloxistatin in human 3D LMS, as highlighted by Schmidt and colleagues for anti-fibrotic properties of gliflozins [54], could generate valuable insights and advance clinical translatability.

To date, no pharmacological intervention currently is approved that directly addresses myocardial fibrosis [10]. Developing effective anti-fibrotic medications has proven a sheer unachievable goal in pharmaceutical research and industry. Considering the high risk for failure and immense cost in drug discovery and development, drug repurposing approaches seem a viable alternative. In a recent study, we could identify and validate the beta-blocker carvedilol as a promising agent to combat fibrosis progression in IPF [55].

## Conclusion

Collectively, the calpain and cathepsin inhibitor aloxistatin demonstrated robust anti-fibrotic and anti-inflammatory effects across multiple cardiac models, including HCFs, rodent LMS, and hiPSC-derived macrophages. By reducing fibroblast proliferation, migration, and ECM formation, and attenuating pro-inflammatory signaling, aloxistatin effectively targets core cellular mechanisms that drive fibrosis progression. Together, these findings strongly highlight aloxistatin’s dual mode of action and underscore its promising potential as a repurposed therapeutic candidate to treat cardiac fibrosis and the maladaptive progression toward heart failure.

## Supplementary Information

Below is the link to the electronic supplementary material.

Supplementary file1 (DOCX 355 KB)

## Data Availability

The generated RNA sequencing data sets are available at EMBL-EBI (https://www.ebi.ac.uk/biostudies/arrayexpress/studies/E-MTAB-16035?key=65fc2fb2-60ba-4cb6-95c3-1bc65e7c30da, https://www.ebi.ac.uk/biostudies/arrayexpress/studies/E-MTAB-16034?key=0c228525-41b8-4d56-957d-3961c212c696).
