# Supplementary material for: Preclinical evaluation of cysteine protease-inhibitor aloxistatin (E64d) for heart failure therapy
Source: J Mol Med (Berl). 2026 Jun 25;104(1):90. doi: 10.1007/s00109-026-02695-5 (PMC13294223; doi:10.1007/s00109-026-02695-5)
Supplement: Supplementary file 1 — Supplementary file1 (DOCX 355 KB) [file 109_2026_2695_MOESM1_ESM.docx]

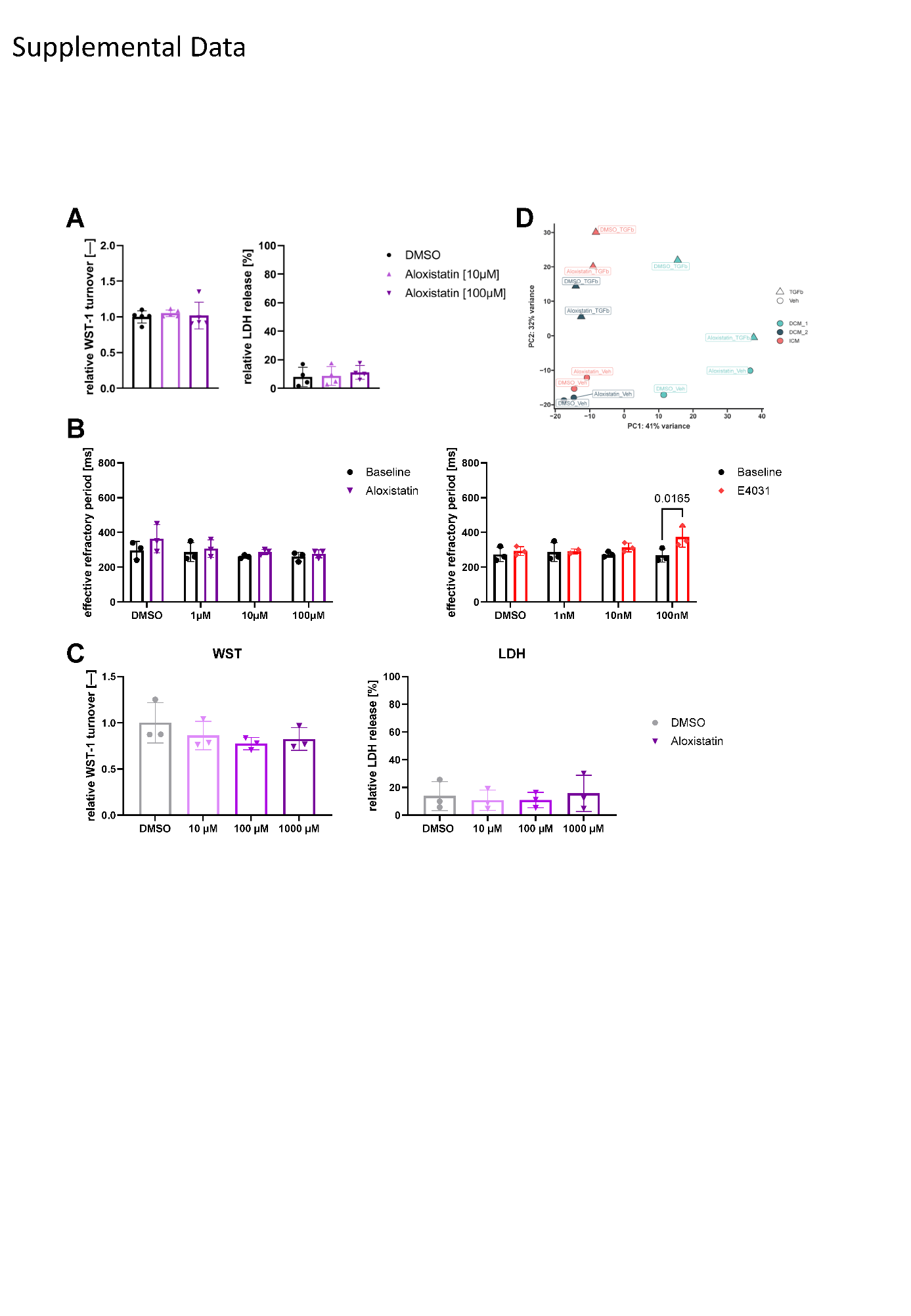


**Supplemental Figure 1:** **(A)** Cellular viability detected by water soluble-tetrazolium salt (WST)-1 and lactate-dehydrogenase (LDH) release of human cardiac fibroblasts after aloxistatin treatment. **(B)** Effective refractory period in porcine living myocardial slices exposed to different concentrations of aloxistatin (left), cardiotoxic agent E4031 (right), or dimethyl sulfoxide (DMSO) control for 15 min measured using dual-pacing strategy coupled with video-based analysis (*n* = 3). **(C)** Viability and toxicity in human precision-cut liver slices after 24 h of treatment with DMSO or aloxistatin at indicated concentrations assessed with WST-1 (left) and LDH assay (right), respectively (*n* = 3). **(D)** Principal component (PC) analysis of transcriptomic data from HCFs exposed to indicated treatments.


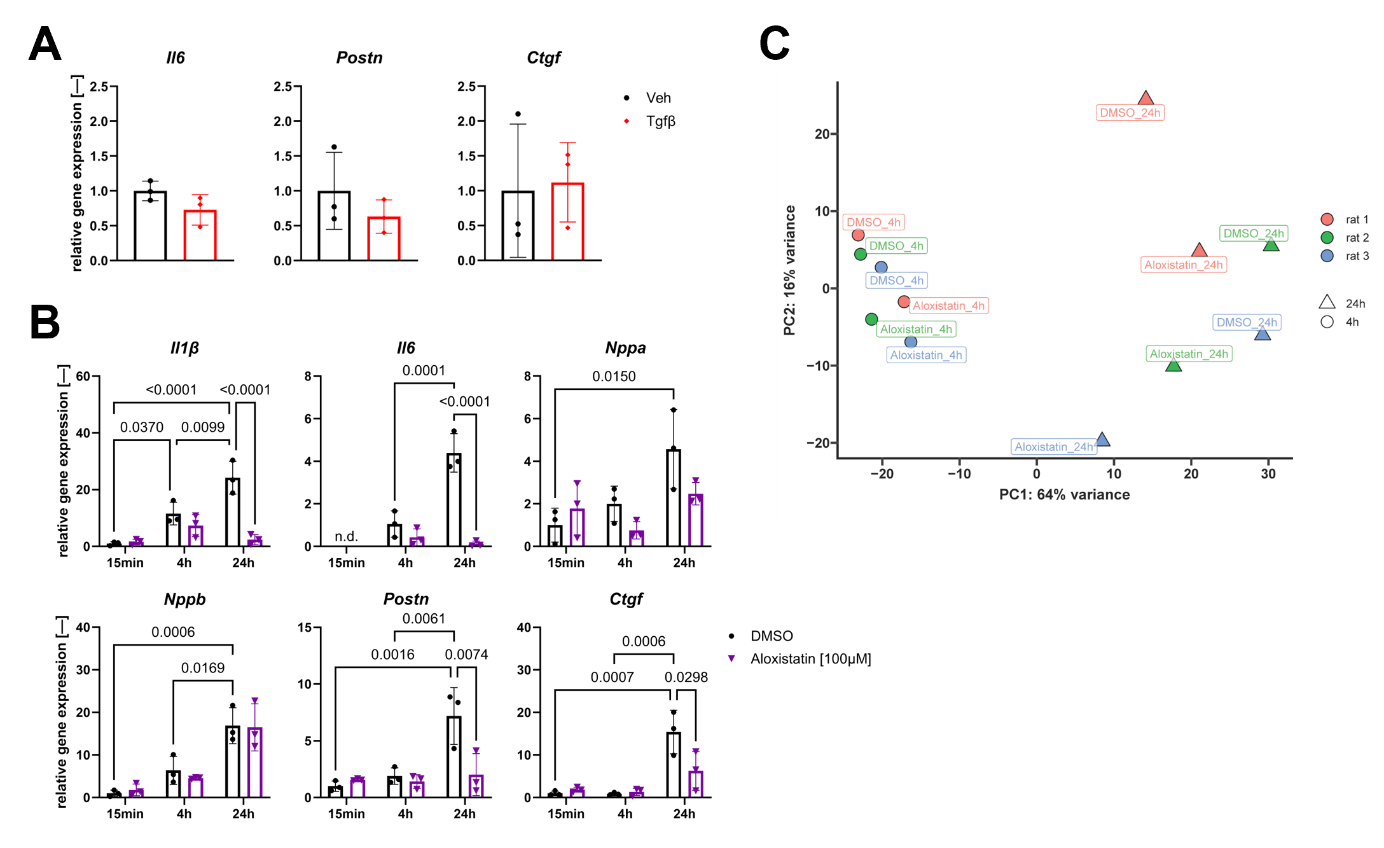


**Supplemental Figure 2:** **(A)** Relative RNA expression of inflammation-associated marker interleukin 6 (*Il6*) and fibrosis-associated markers periostin (*Postn*) and connective tissue growth factor (*Ctgf*) after Tgfβ application to rat LMS (*n* = 3). **(B)** Relative RNA expression of inflammation-associated markers interleukin 1β (Il1β) and interleukin 6 (*Il6*), cardiac stress markers atrial natriuretic peptide (*Nppa*) and B-type natriuretic peptide (*Nppb*), and fibrosis-associated markers periostin (*Postn*) and connective tissue growth factor (*Ctgf*) after LMS cultivation of 15 min, 4 h and 24 h. **(C)** Principal component (PC) analysis of transcriptomic data from rodent LMS exposed to indicated treatments for 4 h and 24 h.

**Supplemental Methods**

**Cardiotoxicity measurement in porcine living myocardial slices**

Acute cardiotoxicity of aloxistatin and E4031 as a positive control was measured in porcine living myocardial slices (pLMS) as described before [1]. In brief, 300 µm thick, 4 × 4 mm wide pLMS were prepared from left ventricular sections of porcine hearts using a vibratome (Model 7000SMZ2, Campden Instruments LTD) and cultivated in a microphysiological system (MPS) [2] for 4 h. Baseline effective refractory period was determined by applying our dual electrical pacing strategy 5 min prior to drug treatment [1]. The measurement was repeated 15 min after drug application to assess acute cardiotoxic events.

**Preparation and treatment of human precision-cut liver slices (hPCLiS)**

Human liver material was obtained from patients (Supplemental Table 1) undergoing partial hepatectomy at Klinikum Region Hannover (KRH) Siloah hospital or Hannover Medical School (MHH), who prospectively gave their consent. Utilization of the tissue for performed experiments was approved by MHH ethics committee and in accordance with relevant guidelines and regulations (applications #3342-2016, Fraunhofer Institut für Toxikologie und Experimentelle Medizin ITEM, and #10855_B0_K_2023, Viszeralchirurgie MHH). Immediately after excision, tissue was placed in ice-cold Belzer UW^®^ storage solution (Bridge to Life Ltd., London, UK). The liver tissue was lamellated into 1 cm thick pieces which were stored in ice-cold Belzer UW^®^ until further processing. For preparation of hPCLiS, cylindrical cores with a diameter of 8 mm were extracted from tissue pieces using a drill [3]. Subsequently, hPCLiS with a thickness of 250 µm to 300 µm were prepared with a Krumdieck tissue slicer MD6000 (Alabama Research and Development, Munford, AL, USA) as described in previous publications [3, 4]. One hPCLiS per well was placed in a 12-well plate and washed with 1 mL Gibco™ William’s E Medium (WME) supplemented with Gibco™ GlutaMAX™-I, 25 mM D-glucose and 50 mg/L gentamicin (all Thermo Fisher Scientific, Waltham, MA, USA) for 1 h at 37 °C under constant orbital rocking at 80 rpm and a humidified atmosphere containing 80% O_2_ and 5% CO_2_. Afterwards, medium was exchanged to WME containing supplements as described before as well as insulin-transferrin-selenium (ITS-solution II, PAN™-Biotech GmbH, Aidenbach, Germany) and aloxistatin or solvent control as indicated, respectively, and hPCLiS were cultivated as outlined above for 24 h.

Supplemental Table 1: Information of liver tissue donors.

| **Biological sex** | **Age [y]** | **Diagnosis** |
| --- | --- | --- |
| **f** | 65 | cholangiocarcinoma |
| **m** | 68 | cholangiocarcinoma |
| **m** | 61 | suspected hemangioma |

**Assessment of viability and toxicity in hPCLiS**

Viability of hPCLiS was measured with water soluble tetrazolium salt (WST)-1 assay (Roche, Basel, Switzerland). Slices were incubated under aforementioned conditions for 1 h in 24-well plates in 475 µL cultivation medium containing 10% (volume per volume [v/v]) WST-1 solution. Afterwards, WST-1 turnover was stopped by lysing the cells through addition of 25 µL 20% (v/v) Triton X100 solution for 15 min. Tissue was homogenized in Lysing Matrix D tubes (MP Biomedicals LLC, Irvine, CA, USA) using a Precellys device (Bertin Technologies SAS, Montigny-le-Bretonneux, France) with the following protocol: 5500 rpm for 20 s twice with 5 s pause. Subsequently, remaining debris were spun down at 10000 ×g for 5 min and optical density at 450 nm (630 nm as reference) was measured in supernatants with a Cytation5 device (Agilent Technologies, Santa Clara, CA, USA).

Toxicity in hPCLiS was assessed with lactate dehydrogenase (LDH) Cytotoxicity Detection Kit (Roche). As for the lysis control, maximal accumulation of LDH in culture medium was achieved by inducing cell necrosis through addition of 1% (v/v) Triton X100 1 h prior to assay start. Culture supernatants were diluted 1:10 in WME and combined with an equal volume of detection reagent solution prepared according to manufacturer’s instructions. After 20 min, optical density at 490 nm (600 nm as reference) was determined with a Cytation5 device.

References

1. Jordan M, Schmieder F, Stucki-Koch A, Polk C, Hansen C, Philipp J, et al. Integrating Heart-on-Chip Technology and 3D Porcine Living Myocardial Slices for Cardiotoxicity Screening. 2025;11:270–3. doi:10.1515/cdbme-2025-0169.

2. Jordan M, Schmieder F, Waleczek FJG, Polk C, Stucki-Koch A, Philipp J, et al. De novo establishment of an ex vivo culture for living myocardial slices applying a microphysiological system – MPSlms. 2024;10:347–50. doi:10.1515/cdbme-2024-2085.

3. Meumann N, Schmithals C, Elenschneider L, Hansen T, Balakrishnan A, Hu Q, et al. Hepatocellular Carcinoma Is a Natural Target for Adeno-Associated Virus (AAV) 2 Vectors. Cancers (Basel) 2022. doi:10.3390/cancers14020427.

4. Granitzny A, Knebel J, Schaudien D, Braun A, Steinberg P, Dasenbrock C, Hansen T. Maintenance of high quality rat precision cut liver slices during culture to study hepatotoxic responses: Acetaminophen as a model compound. Toxicol In Vitro. 2017;42:200–13. doi:10.1016/j.tiv.2017.05.001.
